# Full-Process Temperature Prediction in Multi-Layer Robotic Grinding of High-Manganese Steel Under Limited Online Sensing

**DOI:** 10.3390/s26082422

**Published:** 2026-04-15

**Authors:** Pengrui Zhong, Long Xue, Feng Han, Yong Zou, Jiqiang Huang

**Affiliations:** 1 China Academy of Machinery Science and Technology, Beijing 100044, China; zhongpengrui07@163.com; 2 School of Mechanical Engineering, Beijing Institute of Petrochemical Technology, Beijing 102699, China; hanfeng@bipt.edu.cn (F.H.); zouyong@bipt.edu.cn (Y.Z.); huangjiqiang@bipt.edu.cn (J.H.)

**Keywords:** ZGMn13 high-manganese steel, full-process temperature prediction, multi-layer grinding, thermal accumulation, limited online sensing

## Abstract

Thermal accumulation is a critical constraint in robotic grinding of ZGMn13 high-manganese steel, whereas the variables that can be prescribed or monitored reliably online are often limited to the normal load $F_{z}$, spindle speed n, and feed speed $\nu_{w}$. Most existing studies focus on single-pass conditions or scalar thermal indicators, while full-process near-surface transient temperature histories in multi-layer robotic grinding remains insufficiently addressed. This study presents a full-process near-surface transient temperature histories framework for multi-layer robotic grinding under fixed wheel–workpiece conditions and limited online sensing. Multi-channel near-surface thermal measurements were first reorganized into layer-resolved time-series data. A process-driven thermal surrogate was then constructed from the deployable inputs $\left(F_{z},n,\nu_{w}\right)$, and a recursive temperature-evolution model was developed by incorporating intra-layer thermal retention and interlayer residual-heat inheritance. The proposed formulation predicts the near-surface transient temperature history over successive grinding layers. Experimental results showed clear layer-wise transience and progressive thermal accumulation during multi-layer grinding. Under representative conditions, the proposed framework reproduced the dominant transient structure of the measured full-process near-surface temperature histories, and grouped validation further showed that the recursive formulation preserved more useful history-level information than the reduced baselines within the tested domain. Within the tested operating domain, the predicted histories were further reduced to derived thermal indicators and planning-oriented peak-temperature maps.

## 1. Introduction

ZGMn13 high-manganese steel is widely used in heavy-duty wear-resistant components because of its high toughness and strong work-hardening capability [1,2,3]. Recent studies on austenitic steels have further shown that thermal stability, stacking-fault-energy regulation, and the coupling of deformation mechanisms play important roles in determining their thermomechanical response [4,5]. These characteristics are beneficial in service, but they also increase grinding difficulty and make thermal control critical during finishing and corrective machining [6,7]. In robotic grinding, this issue is further complicated by limited online observability. In practical systems, the variables that can be prescribed or monitored reliably are usually restricted to the normal load $F_{z}$, spindle speed n, and feed speed $\nu_{w}$, whereas tangential force, grinding power, and interface temperature are seldom available for robust online use [8,9,10].

Grinding temperature has long been recognized as a key factor governing surface integrity, residual stress, and thermal damage [11,12,13,14,15]. Existing studies have improved the understanding of grinding heat generation, heat partition, and temperature-field evolution under conventional and high-speed grinding conditions [16,17]. Recent reviews have also summarized advances in grinding temperature theory, sensing, and measurement [18]. Related studies on machining-induced surface integrity under different cooling conditions have likewise highlighted the strong sensitivity of thermal damage to process environment and heat control [19]. However, most available studies remain focused on single-pass analysis, conventional grinding configurations, or laboratory-rich sensing conditions. Full-process thermal evolution in multi-layer robotic grinding has received much less attention.

Recent progress in robotic grinding has mainly concentrated on force control, material-removal modeling, compliance design, tool-condition monitoring, and surface-quality improvement [20,21,22,23,24,25]. These studies have improved the controllability and automation of robotic grinding, but thermal response has rarely been treated as the main modeling target. When temperature is considered, existing studies often focus on condition-specific thermal prediction or derived thermal indicators, rather than a layer-resolved transient history suitable for multi-layer process planning [26,27]. Although such static peak-oriented descriptions are useful for coarse thermal comparison, they do not preserve the within-layer rise-and-decay process, nor do they explicitly represent residual-heat inheritance and progressive thermal accumulation across successive layers.

Data-driven, physics-based, and hybrid approaches have recently been introduced into grinding research [28,29]. These methods show potential for temperature prediction and process optimization, but several limitations remain. First, many models still rely on sensing variables that are not readily available in force-controlled robotic grinding. Second, most temperature-oriented studies have been developed for conventional grinding or for materials other than high-manganese steel [26]. In parallel, recent energy-based modeling studies have further demonstrated the value of specific-energy analysis for clarifying process behavior and improving predictive modeling [30]. Meanwhile, studies on grinding speed effect, material removal, contact behavior, and chip formation [31,32,33,34] have improved the mechanistic understanding of abrasive interaction, but they remain focused mainly on local or single-pass process behavior. Explicit prediction of history-dependent thermal evolution and layer-to-layer heat accumulation in multi-layer robotic grinding therefore remains insufficiently addressed.

These limitations are particularly relevant for robotic grinding of high-manganese steel, where progressive work hardening and repeated abrasive interaction can lead to cumulative thermal loading [1,6]. A practical model for this process should therefore satisfy two requirements: it should operate with deployable process-side inputs, and it should describe the transient thermal response over successive grinding layers.

To address this gap, this study develops a full-process temperature prediction framework for multi-layer robotic grinding of ZGMn13 high-manganese steel under fixed wheel–workpiece conditions and limited online sensing. The central objective is not to reconstruct the interface temperature field, but to predict the near-surface temperature history over successive layers using only the deployable process-side inputs $F_{z}$, n, and $v_{w}$. To this end, multi-channel near-surface thermal observations are first reorganized into layer-resolved time-series data, after which a recursive temperature-evolution model is established by combining a process-driven thermal surrogate with intra-layer thermal retention and interlayer residual-heat inheritance. On this basis, peak-temperature maps and other scalar thermal indicators are treated only as derived outputs of the predicted histories for planning-oriented comparison within the tested operating domain.

The main contributions of this study are as follows.

(1) A full-process, history-resolved thermal prediction problem is formulated for multi-layer robotic grinding under limited online sensing, with the modeling target defined as the near-surface temperature history over successive grinding layers.
(2) A recursive temperature-evolution model is formulated using only the deployable process-side inputs $F_{z}$, n, and $\nu_{w}$, while explicitly incorporating intra-layer thermal retention and interlayer residual-heat inheritance.
(3) (The proposed framework links full-process prediction with process planning by reducing the predicted histories to derived thermal indicators and peak-temperature maps within the tested operating domain.

## 2. Experimental System, Multi-Layer Grinding Protocol, and Thermal Data Construction

### 2.1. Robotic Grinding System and Controllable Process Inputs

The experimental system consisted of an industrial robot, a motor-driven grinding spindle, a force-regulation unit, and a dedicated fixture for the high-manganese steel workpiece. During grinding, the process state was primarily controlled by the spindle speed n, the normal load $F_{z}$, and the feed speed $\nu_{w}$. In the original experimental design, these variables were also used as the principal process factors, as shown in Table 1.

**Figure 1:**
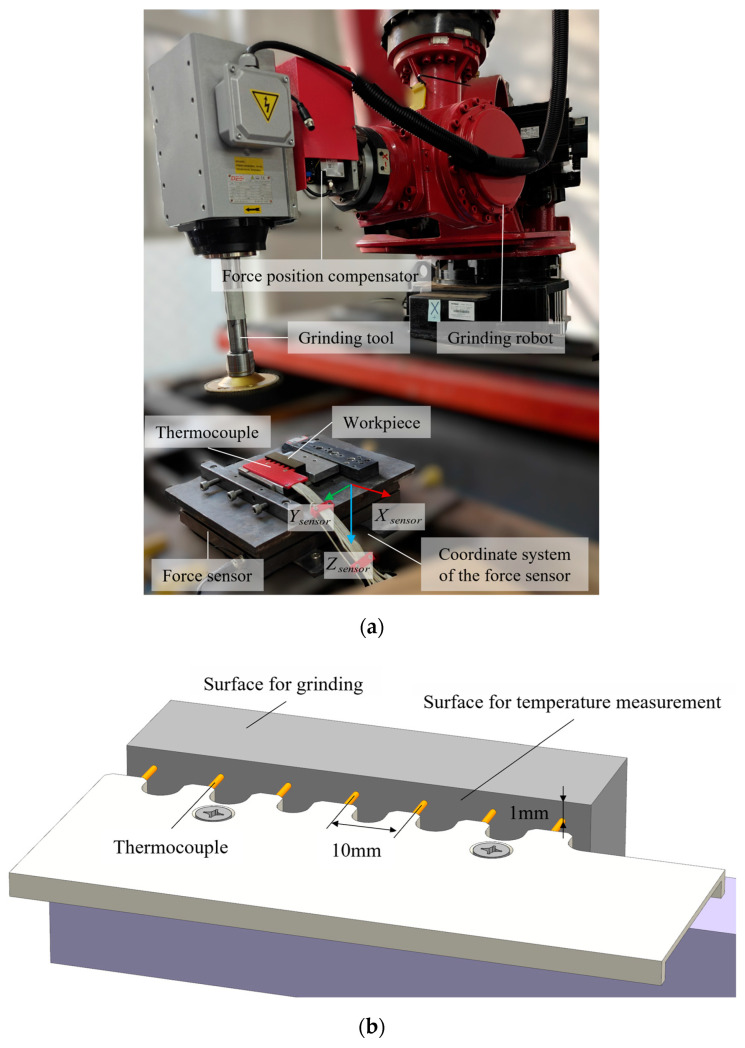
Robotic grinding system, workpiece configuration, and near-surface thermal sensing arrangement. (**a**) Experimental platform; (**b**) Thermocouple layout diagram.

The wheel–workpiece system was treated under fixed geometric conditions. The grinding wheel had a diameter of $D_{w}=$ 125 mm and a grit size of 36, and the wheel–surface angle θ= 2°. Grinding was conducted on ZGMn13 high-manganese steel specimens with a length $L_{w}=$ 75 mm and a width $W_{w}=$ 20 mm, as shown in Figure 1. Under these fixed conditions, the wheel–workpiece contact area was approximated as constant within the tested operating domain.

### 2.2. Multi-Layer Grinding Protocol and Layer-Wise Process Definition

The grinding task was formulated as a multi-layer material-removal process. The target removal was achieved through a sequence of successive grinding layers, and each layer corresponded to one effective pass along the prescribed path over the specimen length. The layer index was defined as l=1,2,…,L, where L denotes the total number of grinding layers in a given experiment.

For the l-th layer, the effective grinding interval was defined by a start time $t_{l,0}$ and an end time $t_{l,e}$. The corresponding layer duration was therefore written as

(1) $$\tau_{l}=t_{l,e}-t_{l,0}\approx \frac{L_{w}}{v_{w}}$$

where $L_{w}=75\;mm$ is the specimen length and $\nu_{w}$ is the feed speed.

Each layer was further divided into an entry stage, a quasi-steady stage, and an exit stage. To support the subsequent dynamic formulation, a normalized local layer-time variable was introduced as:

(2) $$\xi_{l,k}=\frac{t_{k}-t_{l}^{s}}{t_{l}^{e}-t_{l}^{s}},\;\;\;\;0\leq \xi_{l,k}\leq 1$$

where $t_{l}^{s}$ and $t_{l}^{e}$ denote the start and end times of layer l, respectively. This normalized time coordinate provides the basis for defining the temporal modulation term in the subsequent model.

The thermal state of the l-th layer was assumed to inherit the residual heat retained from the (l−1)-th layer. The initial condition of the current layer was therefore written as

(3) $$T_{l}\left(t_{l,0}\right)=T_{amb}+R_{l-1}^{end}$$

where $T_{amb}$ is the ambient temperature and $R_{l-1}^{end}$ represents the residual thermal state at the end of the previous layer. This observation-level dependence provided the basis for the formal recursive initialization introduced later in Section 3.

### 2.3. Variable Roles in Experimental Design and Model Calibration

Thermal response during grinding was recorded by a multi-channel thermocouple array used as an offline observation system. The thermocouples were mounted on the side face of the workpiece, with the sensing locations positioned 1 mm below the grinding surface and adjacent channels spaced 10 mm apart. The objective of the study is therefore not to reconstruct the interface temperature field, but to predict the near-surface temperature evolution that can be measured reliably and used for model calibration and process assessment.

At time t, the observed thermal vector was written as

(4) $$T^{obs}\left(t\right)=\left[T_{1}\left(t\right),T_{2}\left(t\right),\ldots ,T_{C}\left(t\right)\right].$$

For layer l, the corresponding thermal observation sequence was written as

(5) $$T_{l}^{obs}\left(t\right)=\left[T_{\;l,1}\left(t\right),T_{l,2}\left(t\right),\ldots ,T_{l,C}\left(t\right)\right]$$

where C is the number of thermocouple channels. The predictive model therefore relies on process-side inputs alone, while the thermocouple data provide ground-truth thermal evidence for calibration and validation.

### 2.4. Time-Series Synchronization and Construction of Layer-Resolved Thermal Data

The raw experimental records were reorganized into a layer-resolved time-series dataset. All process and thermal signals were first mapped onto a common temporal basis $t_{0},t_{1},\ldots ,t_{K}$, so that the thermal evolution within and across layers could be represented consistently.

After temporal synchronization, the full grinding record was segmented according to layer boundaries. For each layer l, the thermal observations were written as

(6) $$T_{l}(t_{k})=\left[T_{l,1}(t_{k}),T_{l,2}(t_{k}),\ldots ,T_{l,C}(t_{k})\right],{\;t}_{k}\in [t_{l,0},t_{l,e}]$$

The complete thermal dataset was expressed as:

(7) $$T=T_{l,c}\left(t_{k}\right)$$

which served as the basis for model identification and validation.

Several layer-wise thermal-state quantities were defined. The starting and ending temperatures of layer l were obtained through channel aggregation,

(8) $$T_{l}^{start}=A_{c}\left(T_{l,c}\left(t_{l,c}\right)\right),{\;\;\;T}_{l}^{end}=A_{c}\left(T_{l,c}\left(t_{l,e}\right)\right)$$

where $A_{c}\left(\cdot \right)$ denotes a channel aggregation operator, which was defined in this study as the median across thermocouple channels at each time step. The resulting quantity represents an aggregated near-surface thermal response for layer-wise state construction. The residual thermal state at the end of layer l was defined as

(9) $$R_{l}^{end}=T_{l}^{end}-T_{amb}$$

**Figure 2:**
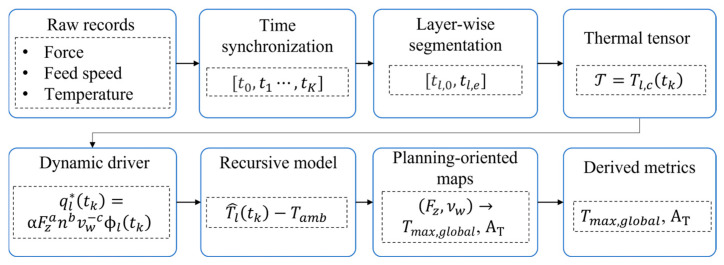
Construction of layer-resolved thermal data and modeling workflow.

This data structure retains both intra-layer thermal evolution and interlayer thermal inheritance, and it forms the basis of the dynamic temperature model developed as shown in Figure 2.

### 2.5. Variable Hierarchy and Modeling Target for Full-Process Temperature Prediction

The variables used in the framework were organized into four categories. The first category was the deployable process input set $\left\{F_{z},n,\nu_{w}\right\}$, which contains the quantities that can be prescribed during robotic grinding. The second category was the fixed system descriptor set $\{D_{w},\theta ,L_{w}$}. The third category was the offline thermal observation set $T_{l,c}\left(t_{k}\right)$ used for calibration and validation. The fourth category comprised the derived dynamic thermal-state variables, such as $\left\{T_{l}^{start},T_{l}^{end},R_{l-1}^{end},T_{l}\left(t_{k-1}\right)\right\}$.

Under this hierarchy, the primary modeling target was defined as the prediction of near-surface temperature histories over successive grinding layers

(10) $$\hat{T_{l}}\left(t_{k}\right)=F\left(F_{z},n,v_{w},l,t_{k},h_{l,k-1}\right)$$

where $h_{l,k-1}=\left[\hat{T_{l}}\left(t_{k-1}\right),R_{l-1}^{end}\right]$ is the dynamic thermal-state vector.

Derived thermal indicators were retained only as quantities obtained from the predicted histories. For example, the global peak temperature was defined as

(11) $$T_{max,global}=\underset{l,k}{\max}\hat{T_{l}}\left(t_{k}\right)$$

and the cumulative thermal exposure was defined as

(12) $$A_{T}=\sum_{l=1}^{L}\int_{t_{l,0}}^{t_{l,e}}\;{\left(\hat{T_{l}}\left(t\right)-T_{ref}\right)}_{+},dt$$

where $T_{ref}$ is a prescribed thermal reference level. These derived thermal indicators were used for evaluation and planning-oriented analysis, but they were not the primary modeling target.

## 3. Dynamic Formulation of Full-Process Temperature Evolution in Multi-Layer Grinding

### 3.1. Problem Definition and Modeling Objective

The study aimed to predict the near-surface temperature evolution during multi-layer robotic grinding using only the deployable process inputs $F_{z}$, n, and $\nu_{w}$. The primary modeling target was not a single scalar thermal indicator, but the prediction of near-surface temperature histories over the full grinding process.

For the l-th grinding layer and the k-th time step, the temperature prediction problem was formulated as

(13) $$\hat{T_{l}}\left(t_{k}\right)=F\left(F_{z},n,v_{w},l,t_{k},h_{l,k-1}\right)$$

where $\hat{T_{l}}\left(t_{k}\right)$ is the predicted near-surface temperature.

Accordingly, the temperature model was formulated as a recursive dynamic system driven by deployable process-side inputs and thermal-state variables.

### 3.2. Dynamic Thermal-Driving Representation

Under the fixed contact geometry considered, the instantaneous thermal driving state was represented by a process-side surrogate

(14) $$q_{l}^{*}\left(t_{k}\right)=\alpha F_{z}^{a}n^{b}v_{w}^{-c}\phi_{l}\left(t_{k}\right)$$

where α is a scaling coefficient, a, b and c are model parameters, and $\phi_{l}\left(t_{k}\right)$ is a normalized temporal modulation term used to represent the entry, quasi-steady, and exit stages within layer l. The surrogate $q_{l}$ represents the effective thermal-driving intensity at time step $t_{k}$. A power-law form was used to describe the monotonic but nonlinear dependence of thermal driving on the deployable process inputs. Under the present fixed wheel–workpiece configuration, increasing $F_{z}$ and n tends to increase thermal input, whereas increasing $v_{w}$ reduces the local thermal residence time and was therefore expressed in inverse form. In this study, $\phi_{l}(t_{k})$ was defined as a fixed piecewise function of the normalized local layer time $\xi_{l,k}$:

(15) $$\phi_{l}(t_{k})=\left\{\begin{array}{cc} \frac{\xi_{l,k}}{\lambda_{in}} & 0\leq \xi_{l,k}<\lambda_{in}\\ 1, & \lambda_{in}\leq \xi_{l,k}\leq 1-\lambda_{out}\\ \frac{1-\xi_{l,k}}{\lambda_{out}} & 1-\lambda_{out}<\xi_{l,k}\leq 1 \end{array}\right.$$

where $\lambda_{in}$ and $\lambda_{out}$ denote the normalized entry-stage and exit-stage fractions, respectively. Accordingly, $\phi_{l}(t_{k})\in [0,1]$, rises from zero during entry, remains unity during the quasi-steady stage, and decays to zero during exit. Under the present formulation, the shape of $\phi_{l}(t_{k})$ was fixed for all layers and conditions, and the process dependence of the instantaneous thermal driving was represented primarily by the deployable inputs $F_{z}$, n, and $v_{w}$. This simplified modulation term was introduced to describe the common rise–hold–decay pattern of the thermal driving state within one grinding layer, while keeping the recursive model compact under limited online observability.

For subsequent recursive modeling, $q_{l}^{*}\left(t_{k}\right)$ was used as the instantaneous thermal input quantity at each time step.

### 3.3. Interlayer Temperature Evolution

The near-surface temperature evolution was described by a discrete recursive model

(16) $$\hat{T_{l}}\left(t_{k}\right)-T_{amb}=\rho \left[\hat{T_{l}}\left(t_{k-1}\right)-T_{amb}\right]+g\left(q_{l}^{*}\left(t_{k}\right)\right)+\eta R_{l-1}^{end}$$

where $T_{amb}$ is the ambient reference temperature, ρ is the intra-layer thermal-retention coefficient, η is the interlayer inheritance coefficient, and $R_{l-1}^{end}$ denotes the residual thermal state at the end of the previous layer.

The mapping from the thermal-driving term to the instantaneous temperature increment was written as

(17) $$g\left(q_{l}^{*}\left(t_{k}\right)\right)=\beta_{1}q_{l}^{*}\left(t_{k}\right)+\beta_{2}{\left(q_{l}^{*}\left(t_{k}\right)\right)}^{2}$$

and the recursive model becomes

(18) $$\hat{T_{l}}\left(t_{k}\right)-T_{amb}=\rho \left[\hat{T_{l}}\left(t_{k-1}\right)-T_{amb}\right]+\beta_{1}q_{l}^{*}\left(t_{k}\right)+\beta_{2}{\left(q_{l}^{*}\left(t_{k}\right)\right)}^{2}+\eta R_{l-1}^{end}$$

The first term describes the retained thermal state from the previous time step within the same layer. The second and third terms describe the current thermal input induced by the process variables. The last term represents the residual heat inherited from the previous layer. Together, these terms allow the model to capture both transient temperature rise within a layer and cumulative thermal buildup across layers.

The initial condition of the l-th layer was written as

(19) $$\hat{T_{l}}\left(t_{l,0}\right)=T_{amb}+\eta R_{l-1}^{end},\;\;R_{0}^{end}=0$$

This recursive form was adopted as the core dynamic model in this study.

### 3.4. Layer-Resolved Observation Model and Calibration Target

The model was calibrated against layer-resolved transient thermal observations. For layer l, the measured multi-channel thermal response was written as

(20) $$T_{l}^{obs}\left(t_{k}\right)=\left[T_{l,1}\left(t_{k}\right),T_{l,2}\left(t_{k}\right),\ldots ,T_{l,C}\left(t_{k}\right)\right]$$

where C is the number of thermocouple channels.

To establish a compact observation target for recursive fitting, the channel-wise observations were aggregated as

(21) $$T_{l}^{obs}\left(t_{k}\right)=A_{c}\left(T_{\left\{l,c\right\}}\left(t_{k}\right)\right)$$

where $A_{c}(\cdot )$ denotes a channel aggregation operator. The median-aggregated transient temperature history was used as the calibration target, so that parameter identification was performed on a compact representative near-surface temperature history rather than on any single channel. The median was adopted to reduce the influence of channel-specific local fluctuations and to provide a more robust near-surface temperature history for recursive calibration.

Parameter identification was performed by minimizing the discrepancy between the predicted and observed temperature histories,

(22) $$L=\sum_{l=1}^{L}\;\sum_{k}\;w_{l,k}{\left[{\hat{T}}_{l}(t_{k})-T_{l}^{obs}(t_{k})\right]}^{2}$$

where $w_{l,k}$ is an optional weighting factor. Global peak quantities were retained only as supplementary evaluation metrics and were not used as the sole regression targets.

### 3.5. Derived Thermal Indicators for Evaluation and Planning

Based on the predicted temperature histories, several derived thermal indicators were defined for model evaluation and subsequent planning-oriented analysis.

The peak temperature of layer l was defined as

(23) $$T_{max,l}=\underset{k}{\max}\hat{T_{l}}\left(t_{k}\right)$$

The full-process global peak temperature was defined as

(24) $$T_{max,global}=\underset{l,k}{\max}\hat{T_{l}}\left(t_{k}\right)$$

To characterize cumulative thermal severity, the thermal exposure of layer l above a reference level $T_{ref}$ was defined as

(25) $$A_{T,l}=\int_{t_{l,0}}^{t_{l,e}}{\left(\hat{T_{l}}\left(t\right)-T_{ref}\right)}_{+}dt$$

where ${\left(\cdot \right)}_{+}$ denotes the positive-part operator. The full-process cumulative thermal exposure was then written as

(26) $$A_{T}=\sum_{l=1}^{L}A_{T,l}$$

The overall modeling chain is summarized as follows: the process inputs $\left(F_{z},n,\nu_{w}\right)$ determine the dynamic thermal-driving term $q_{l}^{*}\left(t_{k}\right)$; the recursive model yields the layer-resolved temperature trajectory $\hat{T_{l}}\left(t_{k}\right)$; and the predicted histories are finally reduced to peak and cumulative thermal indicators for evaluation and planning.

## 4. Experimental Results and Validation

This section evaluates the proposed framework in a progressive manner. First, the measured temperature histories are examined to confirm the presence of layer-wise transience and interlayer thermal accumulation. The recursive model is then identified and assessed against full-process histories, followed by grouped cross-condition comparison with reduced baselines. Finally, planning-oriented peak-temperature maps are derived from the validated model as secondary outputs for process-side thermal comparison within the tested operating domain.

### 4.1. Experimental Evidence of Layer-Wise Thermal Accumulation

**Figure 3:**
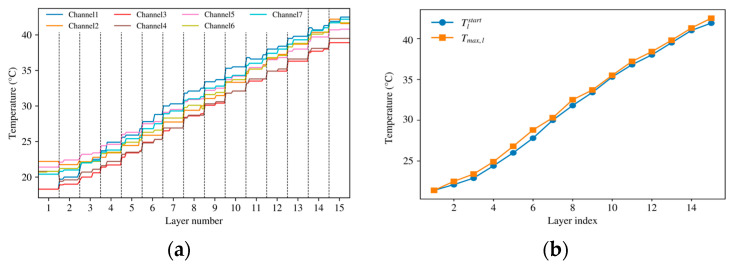
Representative full-process multi-channel temperature histories under multi-layer grinding. (**a**) Temperature history graph; (**b**) Peak temperature graph.

Figure 3 presents representative full-process temperature responses obtained from the multi-layer robotic grinding experiments. The measured histories show a clear layer-resolved transient structure. The thermal level does not return to the initial baseline before the next layer starts, indicating that the thermal process cannot be described as a sequence of independent single-pass events. The multi-channel histories in Figure 3a do not fully overlap, which indicates spatial nonuniformity in the near-surface response. This nonuniformity is consistent with the fact that the thermocouple array provides near-surface observations rather than direct interface-temperature measurements.

Both the starting temperature and the layer-wise peak temperature $T_{max,l}$ increase with layer index, as summarized in Figure 3b. This monotonic upward trend supports the assumption introduced in Section 2 and Section 3 that the thermal state of the current layer is influenced by residual heat retained from the previous layer. In other words, interlayer inheritance is not merely a modeling convenience, but an experimentally observable feature of the process. The purpose of the model is therefore not to reconstruct a detailed spatial temperature field, but to reproduce the aggregated, layer-resolved near-surface temperature history that is stably observable and relevant for process assessment. Accordingly, the model was calibrated against an aggregated layer-resolved temperature history rather than against a channel-specific local temperature.

### 4.2. Identification of the Recursive Temperature-Evolution Model

Parameter identification in this section was performed using the synchronized layer-resolved dataset within the tested operating domain defined in Table 1. In total, 125 sets of experimental data from multi-layer grinding processes were collected. The quality of the thermal observation data is important for recursive model identification. Non-physical abnormalities in the thermocouple records can distort the fitted coefficients and degrade cross-file validation performance; therefore, the experimental data should be carefully screened for instrumental anomalies before model calibration and validation.

**Figure 4:**
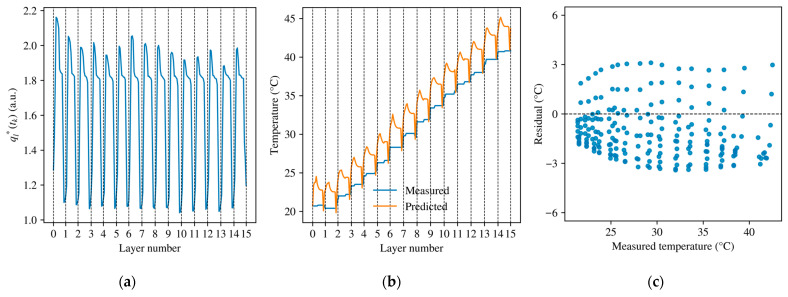
Identification of the dynamic thermal-driving term and recursive temperature-evolution model. (**a**) Thermal-driving history $q_{l}^{*}\left(t_{k}\right)$; (**b**) Temperature Prediction Diagram; (**c**) Residual distribution graph.

The dynamic model was identified according to the formulation in Section 3. Figure 4a shows a representative thermal-driving history $q_{l}^{*}\left(t_{k}\right)$ reconstructed from the deployable process inputs. The sequence preserved the layer-wise structure of the grinding process and provided the instantaneous excitation term for recursive temperature prediction. The calibration result is shown in Figure 4b. The identified model reproduces the dominant structure of the measured full-process temperature history, including the within-layer rise-and-decay pattern and the progressive increase in thermal level over successive layers. This result indicates that the recursive formulation captures the main history-level features of the observed process. The residual distribution in Figure 4c is centered around zero overall, but it is not fully symmetric. Negative residuals are more concentrated over much of the measured-temperature range and tend to reach slightly larger magnitudes than the positive ones. This pattern suggests that, although the present compact formulation captures the dominant transient structure of the full-process response, some systematic mismatch remains, particularly in the detailed reconstruction.

The identified parameters of the thermal-driving term and recursive evolution model are listed in Table 2. These parameters are subsequently used for representative-condition validation, grouped cross-condition comparison, and the derivation of planning-oriented thermal maps. In the present study, the parameters are interpreted primarily by their ability to preserve the observed full-process transient structure under limited deployable inputs. A formal identifiability analysis was beyond the scope of this work. Accordingly, the identified coefficients should be understood as condition-dependent parameters tied to the present modeling form, fixed wheel–workpiece geometry, realized contact state, and calibration target, rather than as directly transferable material constants. Changes in wheel size, workpiece geometry, or material condition would therefore require parameter re-identification.

The identified coefficients are consistent with the recursive formulation. The value of ρ indicates that intra-layer thermal carryover remains non-negligible in the present recursive fit, while its magnitude should be interpreted together with the calibration target, the quality of the thermal observations, and the coupling with the interlayer inheritance term η. The coefficients $\beta_{1}$ and $\beta_{2}$ govern the linear and nonlinear contributions of the thermal-driving term, whereas α, a, b, and c define the scale and input sensitivity of the process-side surrogate.

### 4.3. Time-Resolved Prediction Performance Under Representative Conditions

The time-resolved predictive performance of the proposed model was evaluated using one grinding condition at each spindle speed. The corresponding operating conditions are summarized in Table 3. These representative cases are used for history-level comparison under typical conditions.

**Figure 5:**
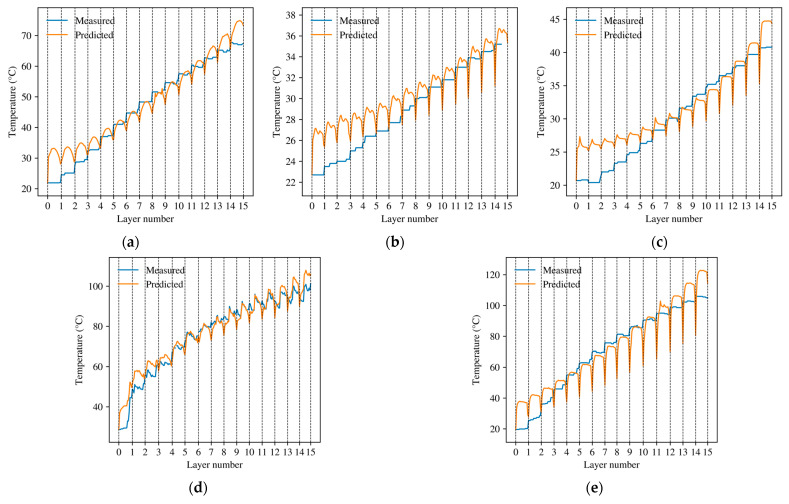
Comparison between predicted and measured full-process near-surface temperature histories under five representative grinding conditions. (**a**) 2500 rpm; (**b**) 3000 rpm; (**c**) 3500 rpm; (**d**) 4000 rpm; (**e**) 4500 rpm.

Figure 5 compares the predicted and measured temperature histories for the five representative conditions. In all cases, the proposed model reproduced the main transient features of the process, including the layer boundaries, the progressive increase in thermal level, and the local rise-and-decay behavior within each layer. The agreement was maintained over the full process rather than only at the maximum temperature.

The quantitative errors are summarized in Table 3. Here, MAE and RMSE were computed from the aggregated full-process temperature histories over all windows of the selected representative file, and peak error was defined as the absolute difference between the measured and predicted global peak temperatures of the same full-process history. Across the five representative cases, MAE ranged from 4.6 °C to 9.6 °C, and RMSE ranged from 5.7 °C to 11.2 °C, indicating that the model preserved the dominant history-level structure of the near-surface thermal response under representative operating conditions.

These representative-condition results support that the proposed formulation can reproduce the main features of the full-process thermal histories across the calibrated spindle-speed range. In Table 3, peak error remained below 10 °C from 2500 rpm to 4000 rpm and increased to 17.8 °C at 4500 rpm, while MAE and RMSE still remained within a moderate range. This pattern indicates that the model is more robust in preserving the dominant history-level structure than in reproducing the exact global peak under the strongest thermal loading. One reason is that the fixed-shape temporal modulation in the present formulation is only a compact approximation of the within-layer thermal-driving profile, and its mismatch may become more evident at higher spindle speeds. Another is that the model was calibrated against the median-aggregated near-surface thermal history rather than against a single local maximum. The present formulation should therefore be interpreted primarily as a predictor of full-process near-surface thermal histories rather than as an exact estimator of every peak-temperature event.

### 4.4. Cross-Condition Validation and Baseline Comparison

The generalization behavior of the model was further examined under file-level leave-one-file-out grouped validation at each spindle speed. In addition to the mean predictor, no-inheritance model, and static peak model, a Jaeger-inspired thermal baseline was introduced as a reduced physics-motivated reference. Using the same deployable inputs $F_{z}$, n, and $\nu_{w}$, its temperature update was written as:

(27) $$T_{l,k+1}^{J}=T_{l,k}^{J}+\Delta t\left(\kappa q_{l}(t_{k})-\lambda \left(T_{l,k}^{J}-T_{amb}\right)\right)$$

where $q_{l}(t_{k})$ is the same process-side thermal-driving term defined in Section 3.2, $T_{amb}$ is the ambient temperature, and κ and λ are identified from the training data.

For each spindle speed, all experimental files recorded under different combinations of normal load and feed speed were treated as independent file-level groups. Each file corresponds to one complete multi-layer grinding experiment under a specific operating condition. Grouped validation was then performed in a leave-one-file-out manner: in each round, one file at that spindle speed was held out as the test set, and the remaining files at the same spindle speed were used for model fitting. This procedure was repeated until every file had served once as the test set, and the grouped MAE, RMSE, and $R^{2}$ reported in Table 4 were obtained by aggregating the prediction errors over all held-out files.

**Figure 6:**
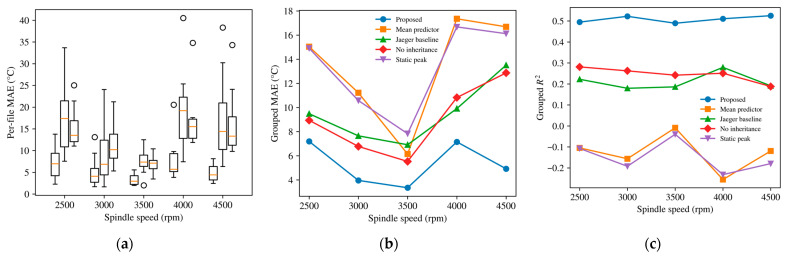
Comparison with baseline models under grouped validation. (**a**) MAE; (**b**) RMSE; (**c**) $R^{2}$.

Figure 6 and Table 4 show that the proposed model maintained positive grouped $R^{2}$ at all five spindle speeds, ranging from 0.49 to 0.53. These values were consistently higher than those of the compared baselines. The no-inheritance model remained positive but lower, at 0.19 to 0.28, and the Jaeger baseline produced similar yet generally slightly lower values of 0.18 to 0.28. By contrast, the mean predictor was non-positive at all spindle speeds (−0.26 to −0.01), whereas the static peak model stayed negative throughout (−0.23 to −0.04).

The grouped MAE and RMSE results show the same overall pattern. Across all five spindle speeds, the proposed model gave the lowest grouped MAE and RMSE. Its advantage over the no-inheritance model was moderate but consistent, whereas the differences relative to the mean predictor and the static peak model were much more evident. The Jaeger baseline remained competitive in part of the tested range, but still did not reach the accuracy level of the full recursive formulation.

The comparison with the mean predictor indicates that the grouped thermal response cannot be represented adequately by an average-history template alone. The comparison with the Jaeger baseline further shows that a simplified heat-input–cooling balance can recover part of the cross-file trend but still leaves a clear gap relative to the proposed model. Compared with the no-inheritance model, the proposed formulation gave lower grouped MAE, lower grouped RMSE, and higher grouped $R^{2}$ at every spindle speed, supporting retention of the full recursive structure.

Taken together, these results indicate that the proposed model preserves more useful history-level information than the tested baselines under cross-file transfer within the tested spindle-speed range. At the same time, this evaluation remains a challenging file-level generalization task under limited process-side observability.

### 4.5. Planning-Oriented Thermal Maps Derived from the Validated Model

**Figure 7:**
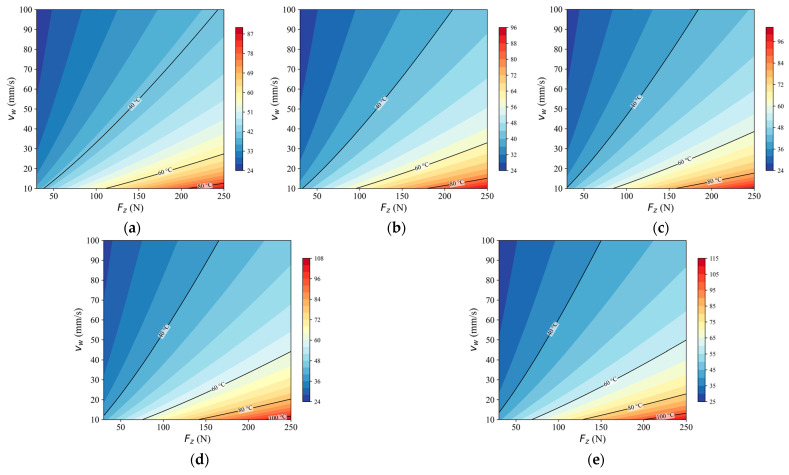
Peak-temperature maps derived from the proposed dynamic model at different spindle speeds. (**a**) 2500 rpm; (**b**) 3000 rpm; (**c**) 3500 rpm; (**d**) 4000 rpm; (**e**) 4500 rpm.

Based on the identified model, peak-temperature maps were generated to illustrate the planning-oriented output of the proposed framework. Figure 7 presents the maps of $T_{max,global}$ on the $\left(F_{z},{\;\nu }_{w}\right)$ plane at fixed spindle speeds.

Across all spindle speeds, the predicted peak temperature increased with normal load and decreased with feed speed. The gradient along the $F_{z}$ direction was consistently positive, whereas the gradient along the $\nu_{w}$ direction was negative. The overall contour pattern was preserved across spindle speeds, although the absolute thermal level and contour spacing varied with n.

These maps were derived from the predicted full-process temperature histories generated by the recursive model. They provide a compact representation of peak thermal severity within the deployable process-input space.

In practical use, these maps can serve as a pre-planning aid for selecting process parameters before a multi-layer grinding job. For a given spindle speed, the operator or robot programmer may first identify the region associated with a lower predicted peak temperature, and then choose a feasible combination of normal load $F_{z}$ and feed speed $\nu_{w}$ within that region according to productivity and process constraints. In general, the maps indicate that reducing $F_{z}$ or increasing $\nu_{w}$ tends to lower the predicted thermal severity. The present maps are therefore intended for parameter screening and planning-oriented comparison within the tested operating domain, rather than as a direct real-time decision rule or a universal thermal-damage threshold.

## 5. Discussion

The results indicate that multi-layer robotic grinding should be treated as a history-dependent thermal process rather than as a sequence of independent single-pass events. The measured thermal histories showed clear layer-wise transience, and both the starting temperature and the layer-wise peak temperature increased with layer index. This observed trend justifies the use of a recursive, history-resolved formulation for the present problem.

The representative-case results in Table 3 show that the proposed model can reproduce the dominant transient structure of the full-process near-surface temperature histories under typical operating conditions. The agreement remained stable at the history level across most tested conditions, whereas the peak-temperature error became more pronounced under the strongest thermal loading. This result suggests that the present model is more reliable as a predictor of full-process temperature histories than as an exact estimator of every peak-temperature event.

Table 4 provides a stricter cross-file evaluation on unseen files at each spindle speed. Under this setting, the proposed model consistently achieved the lowest grouped MAE and RMSE and the highest grouped $R^{2}$ among the compared models. These results indicate that the full recursive formulation preserves more useful history-level information than the reduced baselines, although prediction on unseen files remains challenging under limited process-side observability.

The present results should not be interpreted as evidence of broad transferability beyond the tested operating domain. In the current formulation, the identified coefficients are tied not only to the material response, but also to the realized contact geometry, thermal-driving scale, and aggregated near-surface temperature-history definition under the present setup. Changing the wheel size, workpiece geometry, contact condition, or material grade would therefore require parameter re-identification rather than direct parameter transfer. Within this scope, the framework should be interpreted as a compact, process-specific predictor of full-process near-surface temperature histories using limited process-side inputs, rather than as a universal thermal model.

## 6. Conclusions

This study presented a full-process temperature prediction framework for multi-layer robotic grinding under fixed wheel–workpiece conditions and limited online sensing. The main conclusions are as follows:

(1) The measured near-surface thermal histories showed clear layer-wise transience and progressive interlayer thermal accumulation, indicating that multi-layer grinding is a history-dependent thermal process.
(2) Using only the deployable process-side inputs, the proposed recursive formulation reproduced the dominant structure of the measured full-process near-surface temperature histories under representative operating conditions.
(3) Under file-level leave-one-file-out validation at each spindle speed, the proposed model consistently outperformed the compared baselines in grouped MAE, RMSE, and $R^{2}$, although prediction on unseen files remained challenging under limited process-side observability.
(4) The predicted temperature histories can be further reduced to derived thermal indicators and planning-oriented peak-temperature maps in the deployable input space. Within the tested operating domain of the present study, these outputs provide a practical aid for parameter screening and planning-oriented comparison before multi-layer grinding.

Overall, the proposed framework provides a compact and practically deployable approach for predicting representative near-surface temperature histories in multi-layer robotic grinding under limited online sensing.

## Figures and Tables

**Table 1:** Primary process inputs and tested levels.

Level	A: Spindle Speed *n* (rpm)	B: Normal Load *F_z_* (N)	C: Feed Speed $\nu_{w}$ (mm/s)
1	2500	50	10
2	3000	100	25
3	3500	150	40
4	4000	200	55
5	4500	250	70

**Table 2:** Identified parameters of the thermal-driving term and recursive temperature-evolution model.

Parameter	α	a	b	c	ρ	η	$\beta_{1}$	$\beta_{2}$
Value	0.17	0.294	0.24	0.26	0.42	0.59	−2.93	1.96

**Table 3:** Time-resolved prediction versus measurement under representative grinding conditions.

Spindle Speed	$F_{z}$	$\nu_{w}$	MAE	RMSE	Peak Error
2500	175	32	5.6	6.8	9.5
3000	90	52	4.6	5.7	8.6
3500	130	55	5.1	6.3	9.7
4000	90	9	4.8	5.9	8.9
4500	180	20	9.6	11.2	17.8

**Table 4:** Comparison with baseline models under grouped validation.

Spindle Speed		Proposed	Mean Predictor	Jaeger Baseline	No Inheritance	Static Peak
2500	MAE	7.2	15	9.5	8.9	14.9
	RMSE	9	18.1	10.5	10	18.1
	$R^{2}$	0.49	−0.1	0.22	0.28	−0.11
3000	MAE	4	11.2	7.6	6.8	10.6
	RMSE	6.4	14.3	11.5	15.5	14.5
	$R^{2}$	0.52	−0.16	0.18	0.26	−0.19
3500	MAE	3.3	6.1	6.9	5.5	7.8
	RMSE	4.1	8	8.3	7.6	8.7
	$R^{2}$	0.49	−0.01	0.19	0.24	−0.04
4000	MAE	7.1	17.4	9.9	10.8	16.7
	RMSE	9.8	21.2	13.7	14.3	21
	$R^{2}$	0.51	−0.26	0.28	0.25	−0.23
4500	MAE	4.9	16.7	13.5	12.9	16.1
	RMSE	7.1	21.2	16.8	15.4	21.7
	$R^{2}$	0.53	−0.12	0.18	0.19	−0.18

## Data Availability

Data are contained within the article.
